# Salivary diagnostics: opportunities and challenges

**DOI:** 10.7150/thno.100600

**Published:** 2024-10-21

**Authors:** Yuxin Li, Yourong Ou, Kexin Fan, Guozhen Liu

**Affiliations:** Integrated Devices and Intelligent Diagnosis (ID2) Laboratory, CUHK(SZ)-Boyalife Joint Laboratory for Regenerative Medicine Engineering, Biomedical Engineering Programme, School of Medicine, The Chinese University of Hong Kong, Shenzhen, 518172, China.

**Keywords:** Salivary diagnostics, non-invasive detection, biomarkers, result standardization, point-of-care testing

## Abstract

Saliva contains a diverse array of biomarkers indicative of various diseases. Saliva testing has been a major advancement towards non-invasive point-of-care diagnosis with clinical significance. However, there are challenges associated with salivary diagnosis from sample treatment and standardization. This review highlights the biomarkers in saliva and their role in identifying relevant diseases. It provides an overview and discussion about the current practice of saliva collection and processing, and advancements in saliva detection systems from *in vitro* methods to wearable oral devices. The review also addresses challenges in saliva diagnostics and proposes solutions, aiming to offer a comprehensive understanding and practical guidance for improving saliva-based detection in clinical diagnosis. Saliva diagnosis provides a rapid, effective, and safe alternative to traditional blood and urine tests for screening large populations and enhancing infectious disease diagnosis and surveillance. It meets the needs of various fields such as disease management, drug screening, and personalized healthcare with advances in saliva detection systems offering high sensitivity, fast response times, portability, and automation. Standardization of saliva collection, treatment, biomarker discovery, and detection between different laboratories needs to be implemented to obtain reliable salivary diagnosis in clinical practice.

## Introduction

Saliva, along with other oral fluids such as gingival crevicular fluid and combined secretions of minor salivary glands, play a vital role in supporting the health of both soft and hard tissues in the oral cavity. Biocomponents found in oral fluids include proteins and related molecules, nucleic acid components, extracellular vesicles (EVs), and endogenous and exogenous metabolites 1,2,3. Thus, saliva is recognized as a mirror reflecting the health status of the body. So far, it has been clinically possible to diagnose a range of diseases through saliva 4,5, including infectious diseases, genetic disorders, metabolic diseases, and immunologic diseases. This mainly relies on the role of biomarkers in saliva in the diagnosis, prognosis, prediction, and monitoring of diseases. For example, saliva samples can indicate the concentration of drugs in the body, allowing for drug intake to be monitored and controlled. This helps maintain drug levels within the optimal treatment range, enabling personalized treatment for each patient 6,7. It is noninvasive and painless to collect saliva samples; the possibility of using salivary biomarkers for detecting systemic diseases may constitute a promising opportunity to implement strategies for diagnosing and managing patients affected by chronic diseases, thereby limiting the risks associated with more invasive surgeries 8. Several diagnostic tests that use saliva or oral fluids for the detection of SARS-CoV-2 have received authorization for emergency use from the U.S. Food and Drug Administration 9,10. Since then, research on saliva detection has become a focal point in analytical science, highlighting the numerous benefits of salivary diagnostics in healthcare, especially in a point-of-care scenario.

**Figure 1:**
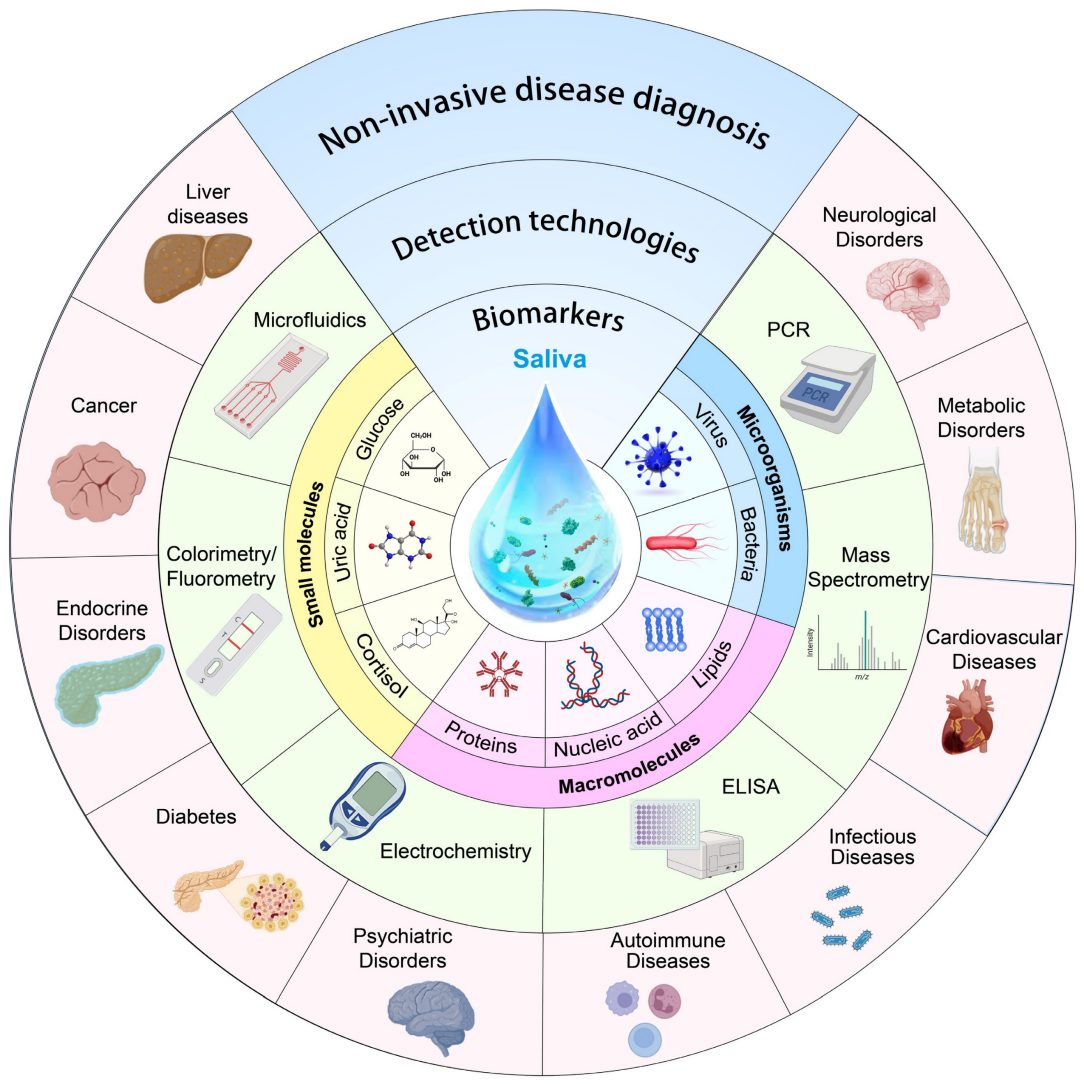
Schematic drawing highlighting the review content.

Looking ahead, the market for salivary diagnosis can only be expected to grow as new biomarkers are discovered and validated. So far, researchers have made unremitting efforts to review saliva diagnosis: Liao *et al.* discussed saliva analysis and its applications in various medical diagnosis and monitoring 11, Song *et al.* provided a comprehensive summary of saliva biomarkers and their testing 12, and Giovanni *et al.* primarily addressed methods for evaluating saliva biomarkers and protocols for saliva collection 8. However, there are several challenges associated with saliva diagnosis including identifying biomarkers unique to a given health condition, guaranteeing uniformity in the collection and preservation of saliva samples, and evaluating the specificity and sensitivity of salivary tests. Being different from the reported work, this review (**Figure 1**) aims to overview advances of key factors in saliva diagnosis, including biomarkers in saliva, current cutting-edge saliva detection techniques, and clinical saliva sample pre/post-processing methods. The potentials, challenges, and perspectives of saliva diagnosis in disease screening, management, and prevention are the primary focuses of this review.

## Saliva components and biomarkers

Saliva is secreted by glands in the oral cavity, and salivary gland cells produce mucous and serous saliva from plasma cells and mucous cells, respectively. Because salivary glands primarily secrete fluid originating from intercellular fluid, the final saliva composition in the oral cavity also incorporates elements from blood 13, which means the composition of saliva not only reflects local oral health conditions but also systemic physiological states. This greatly increases the possibility of saliva as a non-invasive, fast, and convenient biological fluid to replace blood samples for diagnosing diseases in clinical practice. **Table 1** lists comprehensive information of the components in saliva, and some salivary biomarkers used for the clinical diagnosis of diseases and relevant cancers.

Saliva includes 99% water, 0.5% organic and inorganic materials, as well as a significant number of biological components. Organic matter is mainly a variety of proteins and hormones that play different roles, and inorganic matter is mainly Na^+^, K^+^, Cl^-^, and Ca^2+^. According to Johan's work, salivary K^+^ concentration is higher than its plasma concentration (25 *vs* 4 mmol/L), while salivary Na^+^ concentration is lower than that in plasma (2 *vs* 145 mmol/L) 14. Unlike blood plasma, which has 99% of its protein content dominated by 22 proteins, salivary protein composition is more diverse, with its top 20 proteins comprising only 40% of the total 15. Numerous enzymes were found in saliva, such as carbonic anhydrase, lysozyme, salivary amylase, phosphatase, kallikrein, and peroxidases, some of which have been utilized as biomarkers for diagnosing various diseases 16. Hathama *et al.* measured the activity of alkaline and acid Ribonucleases (RNases) enzymes in the saliva of patients with benign and malignant breast tumors, as well as healthy controls. The results showed a highly significant elevation of RNase activity in the saliva of the breast cancer group compared to the benign tumor and control groups 17. Patients with oral cancer had elevated levels of lactate dehydrogenase, a crucial enzyme in the metabolism of cellular energy, which suggested great glycolytic activity and tissue damage 18. Due to dysregulated activity, salivary peroxidase, an antioxidant enzyme, may also contribute to the development of oral cancer and may have an impact on antioxidant defense mechanisms 19. Nucleic acids secreted by cells or microorganisms in the mouth constitute a group of key biomarkers in saliva, closely related to diagnosing various diseases and cancer. In addition, nano-sized EVs were discovered in saliva, which are derived from almost any type of cells in the organism and are present in all body fluids. They carry a variety of finger-print biochemicals, such as ribosomal RNA, microRNA, long non-coding RNA, DNA, and proteins, which participate in the information exchange of recipient cells and regulate the function of recipient cells. Therefore, salivary EVs can be considered as biomarkers for various diseases, including cancer, amyotrophic lateral sclerosis, and asthma 3,20. However, high protein and viscous material concentrations in human saliva provide difficulty to the extraction of EVs because they can encase and disguise the EVs during extraction 21. Saliva can also reflect hormone levels in the human body, such as steroid hormones 22, and closely matches blood in electrolyte and trace element levels 23.

Due to the fact that the content of most biomarkers in saliva is lower than that in blood, so several tactical methods are used to address the variations in concentrations of blood components in saliva with the goal of improving its diagnostic potential, such as critical protocols of creating multiplexed assays, standardizing collection procedures, and establishing a correlation between blood levels and salivary concentrations. It also requires extremely sensitive detection techniques 24,25, and entails normalizing test results to stable salivary components and thus reduce variability 8. Advances in these protocols will significantly enhance the accuracy and capability of saliva diagnostics as a competitive test sample substitute for blood, especially in situations involving regular or non-invasive monitoring, which will be discussed in Section 5.

### Protein and peptide biomarkers

Protein and peptide biomarkers are crucial for diagnosing diseases, monitoring disease progression, and evaluating responses to treatment. Their levels offer a window into the molecular mechanisms underlying various health conditions. As shown in Table 1, among various proteins and peptides in saliva, matrix metalloproteinases (MMPs) and prostate-specific antigen (PSA) are two most studied salivary biomarkers. MMPs are responsible for breaking down extracellular matrix proteins, which is a prerequisite for many biological processes such as cell division, invasion, and metastasis. They are involved in various physiological processes, including tissue repair, angiogenesis, and embryonic development, as well as pathological conditions such as inflammation, cancer, and cardiovascular diseases. Overactive MMPs can help tumor cells traverse the extracellular matrix and basement membrane for invasion and metastasis while promoting tumor growth and angiogenesis, and thereby providing necessary nutrients and oxygen for tumors. As potential diagnostic and prognostic biomarkers for oral cancer, MMP-1, MMP-2, MMP-10, and MMP-12 levels were significantly increased compared with those of other groups. Especially MMP-1 cutoff values reach 199 pg/mL 81. A study on salivary MMP-9 levels in subjects with oral squamous cell carcinoma (OSCC), oral potentially malignant disorders (OPMD), tobacco users, and healthy controls found that OSCC and OPMD groups had significantly higher mean MMP-9 levels than that with tobacco habits and control groups 82. The study suggested that salivary MMP-9 could be a useful, non-invasive biomarker in the diagnosis, treatment, and management of OSCC and OPMD. Another study observed that salivary MMP-9 could be a critical diagnostic and prognostic biomarker for OSCC (analysis of covariance and multivariable linear regression, p<0.05), and MMP-9 decreased from 588.15 to 131.80 ng/mL after tumor surgery in 9 months (p<0.05) 83. PSA is an enzyme produced by the prostate gland and serves as a key protein biomarker in cancer diagnosis. Elevated levels of PSA in the blood can indicate conditions such as prostate cancer, benign prostatic hyperplasia, or prostatitis 84. A correlation has been identified between levels of PSA in salivary and serum of patients with prostate adenocarcinoma 85. Nevertheless, research by Elgamal *et al.* demonstrated the detectability of PSA in both normal and cancerous tissues remote from the prostate, indicating that exclusive reliance on PSA for diagnosis may lead to an overestimation of prostate cancer prevalence 86. It is notable that additional molecular biomarkers, such as the prostate health index, prostate cancer antigen 3, and kallikrein-related peptidase, are available and can complement PSA testing to refine diagnostic specificity and sensitivity 87. In this context, Khan *et al.* have developed an electrochemical biosensor with the capability to detect salivary PSA with high sensitivity (40 fg/mL) and selectivity 88. Contini *et al.* identified concentrations of proteins and peptides such as S100A8, S100A9, and cystatin B variants elevated in the saliva of Alzheimer's patients, indicating their roles in maintaining oral cavity homeostasis and providing neuroprotection 89.

There are many other protein biomarkers in saliva (Table 1), such as CRP and TNF-α produced by the human body during inflammation, which have been extensively studied and can be quickly detected 90,91. In summary, the abundant protein biomarkers in saliva provide new possibilities for early diagnosis and monitoring of systemic diseases, and some biomarkers are effective indicators for diagnosing oral and systemic diseases. With more advanced technologies of proteomics, we are expected to have more protein biomarkers discovered in saliva.

### Nucleic acid biomarkers

Like proteins, salivary nucleic acid biomarkers are essential for non-invasive diagnosing and monitoring both infectious and non-infectious diseases. First of all, saliva is recognized as a viable diagnostic specimen for detecting common respiratory viruses and can be used to detect viral nucleic acids, including those of SARS-CoV-2, human immunodeficiency virus (HIV), and human papillomavirus (HPV) 92. Additionally, circulating tumor DNA (ctDNA) in saliva has emerged as a promising biomarker for diagnosing various diseases, particularly cancers. Salivary ctDNA is particularly effective in detecting HPV-related head and neck squamous cell carcinomas (HNSCC). Studies have shown that HPV ctDNA can identify patients with HNSCC and monitor effectiveness of treatment 93. Salivary miRNA was discovered to be useful in diagnosing OSCC patients since much higher levels were observed in diseased cohort groups 94. Similarly, research on salivary exosomal miRNAs in OSCC patients identified different expression profiles, suggesting they may be potential diagnostic biomarkers of OSCC 95. Beyond miRNA, other salivary biomarkers for OSCC diagnosis have been explored, including cytokines, cell-free RNA (cfRNA) 96. Proteases like KLK5 and uPA 97, and salivary metabolites 98, each contributing to the expanding landscape of non-invasive diagnostic biomarkers for OSCC. Breast cancer gene (BRCA) mutation is another potential salivary biomarker for diagnosing cancer, particularly breast cancer and ovarian cancer 99. Research by Vanstone *et al.* demonstrated the feasibility of detecting BRCA1 and BRCA2 mutations in saliva, offering a reliable, non-invasive, and cost-effective alternative to traditional blood tests 100. Moreover, patients with breast cancer had higher levels of salivary BRCA1 methylation, which may be a useful biomarker 101. Studies have explored salivary DNA methylation and abnormal promoter hypermethylation in genes as diagnostic tools for head and neck cancer, highlighting specific genes like KIF1A and EDNRB as potential biomarkers 102,103. This body of research underscores the diagnostic promise of nucleic acid-based salivary biomarkers, particularly through the lens of epigenetic modifications. All in all, saliva represents a rich source of DNA and RNA biomarkers, and nucleic acid biomarkers represent a promising frontier in precision medicine, offering valuable insights into disease mechanisms and enabling more effective diagnosis and treatment strategies. Technological advancements will further amplify their clinical utility and application across various medical fields by providing enhanced detection sensitivity and specificity.

### Hormonal biomarkers

Saliva contains various hormones that play significant roles in both local and systemic physiological processes. Hormones such as cortisol, human chorionic gonadotropin (hCG), and insulin in saliva have been found to correlate strongly with their levels in serum, indicating saliva analysis provides a non-invasive method for the accurate monitoring of hormone levels in the human body. Cortisol is a stress hormone, indicating the presence and magnitude of stress or stress-related conditions 104. Salivary cortisol levels vary from 0.5 to 50 ng/mL 105 and can be used to screen for Cushing's syndrome 59. Sharma *et al.* introduce a commercial graphene foam (GF) electrode modified with 1-pyrenebutyric acid N-hydroxysuccinimide ester (PBASE-NHS) to create an ultra-sensitive biosensor for detection of cortisol directly in human saliva, with a detection limit of 0.24 fg/mL 106. Estrogen plays a vital role in the normal physiology of the female breast as well as in the pathogenesis of breast cancer. Levels of estrogen and progesterone in saliva were both 0.1-10 ng/mL 12. Certain forms of estrogen can be metabolized into compounds that can form DNA adducts and potentially lead to mutations. Estrogen-receptor-positive breast cancers, which make up a significant proportion of all breast cancers, rely on estrogen for their growth. The study investigated the presence of cancer antigen 15-3 (CA 15-3) and c-erbB-2 in the saliva of healthy women and found indications of breast cancer antigen 107. Mohammed *et al.* proposed a two-stage classification system for breast tumor biomarkers in a sample of Iraqi women, which included saliva-based attributes 108. But even if salivary estrogen is found to have some diagnostic value, it is likely to be used in conjunction with other biomarkers. Mahajan pointed out that salivary hCG can be used as a biomarker for early detection of pregnancy, and is usually detectable at about 3-4 weeks of pregnancy. hCG levels continue to increase throughout the pregnancy 109. Lei *et al.* measured the concentration range of salivary hCG from 0.3 mIU/mL to 0.8 mIU/mL by using graphene-based immunoassay chemiluminescence resonance energy transfer 110. Insulin is a peptide hormone produced by the beta cells of the pancreas. It plays a crucial role in regulating blood glucose levels in the body 111. Myette-Côté *et al.* revealed that the insulin concentration in fasting saliva was around 30% of that in plasma. They also concluded that regardless of the time of the day, the trend of post-meal salivary and plasma insulin reactions is similar, with a lag of about 30-45 min between saliva and plasma insulin reactions 112. For non-invasive insulin measurement, Lin *et al.* developed a method for hypersensitive detection of salivary insulin with a sensitivity of 10 fg/mL based on the CRISPR/Cas system, which has huge advantages in analytical and clinical investigations 113. They provide a promising method for salivary insulin detection with high sensitivity toward non-invasive diabetes/prediabetes early diagnosis. So far, salivary hormone testing can aid in diagnosing and managing conditions related to hormonal imbalances, such as reproductive health issues, and metabolic disorders.

### Metabolite biomarkers

Salivary metabolites have been identified as potential biomarkers for a range of diseases, including HNSCC, OSCC, heart failure, and type 2 diabetes. Uric acid (UA) is a molecule that is produced by the breakdown of purines in the body. Elevated UA levels can be indicative of increased purine metabolism or decreased excretion, which could be associated with OSCC, and emotional disorders (such as anxiety and mood disorders). Salivary UA levels have been investigated as a noninvasive biomarker of metabolic syndrome and may be a useful surrogate for blood testing of UA levels, as a linear relationship between salivary and serum UA levels has been observed 114. Anitha *et al.* found that the combined profile of UA levels in serum and saliva can be used as a diagnostic profile in oral squamous cell carcinoma patients 115. Goh *et al.* found that the various classes of metabolites (from small fatty acids like chain amino acids or fatty acids to larger glycolipid and carbohydrate metabolites) showed considerable promise for minimally invasive diagnosis, especially for HNSCC and OSCC 116. Recently, a microfluidic paper-based analytical device for the non-invasive detection of salivary UA was developed based on digital quantification of color intensity by MATLAB code, and its parallelism was validated in clinical samples 117,118. The salivary UA assay sensitivity was 50 µM which is significantly higher than the sensitivity of market available test strip based on electrochemical detection of UA in fingerpick blood (150 µM). It promises non-invasive disease screening by detection of salivary metabolites. Sanghoon *et al.* investigated the use of electrochemical biosensors to detect lactate in saliva and confirmed a significant increase in lactate levels in saliva during and after exercise 119. Although salivary lactate shows promise as a measure of anaerobic capabilities, variations in saliva composition and sample techniques prevent it from being fully endorsed as a substitute for blood lactate 120. Furthermore, research has demonstrated the potential of salivary lactate as a non-invasive biomarker for the surveillance of heart failure, with encouraging findings suggesting its usefulness in clinical settings 121. For the purpose of diagnosing, screening, and tracking type 2 diabetes mellitus, salivary glucose has been investigated as a non-invasive substitute for blood glucose 122. Due to the non-invasive nature of saliva collection, studies have demonstrated that salivary glucose levels are comparable to blood glucose levels in the diagnosis and monitoring of type 2 diabetes 123. Salivary metabolites can provide insights into the underlying biological pathways involved in diseases. This broad applicability highlights their potential in early disease detection and monitoring.

### Others

A growing amount of research highlighted the significance of saliva as a medium for detecting disease biomarkers, particularly in cancer 124,125. Palanisamy *et al.* found that saliva contains exosomes released by salivary glands and other oral cells, which carry proteins and functional mRNA. Wong *et al.* discovered that exocrine microbubbles from breast cancer could alter the composition of saliva by interacting with salivary gland cells, suggesting discriminatory profiles of salivary biomarkers for diseases developing distally from the oral cavity. Wong's team further confirmed the critical role of tumor-derived exosomes in forming specific salivary transcriptome biomarkers for pancreatic cancer, utilizing an *in situ* homologous pancreatic cancer mouse model 126. In another study of children with chronic kidney disease, researchers observed that compared with the control group, the levels of pro-inflammatory factors, anti-inflammatory factors, T helper cells, cytokines, growth factors, and macrophages in the unstimulated saliva of children with chronic kidney disease were increased or decreased to varying degrees.^127^. Additionally, one research also delved into the role of circulating tumor cells (CTCs) and circulating tumor DNA in saliva 128. CTCs, which shed from primary tumors into body fluids like blood and saliva, though rarer in saliva, present a promising avenue for non-invasive cancer detection. Patel *et al.* explored combining salivary mRNA and CTC levels for detecting head cancer with high accuracy 129. Despite the majority of CTC research focusing on blood samples, the investigation into saliva as a source of CTCs is growing, with the potential for non-invasive monitoring and diagnosis of cancer. Salivary redox biomarkers are emerging as valuable tools for diagnosing and monitoring various health conditions, including chronic diseases. Studies on salivary redox biomarkers have been conducted in relation to systemic diseases such as type 2 diabetes mellitus, periodontitis, oral malignancies, and Sjögren's syndrome 130. These biomarkers provide insights into the oxidative stress status within the body by measuring specific molecules and enzymes in saliva. Neurodegenerative disorders (NDDs) such as Alzheimer's, Parkinson's, and Huntington's disease can be diagnosed and their prognosis can be determined using salivary redox biomarkers, which have shown promise as non-invasive indicators 131. Heme oxygenase-1 (HO-1) is one salivary redox biomarker that has demonstrated diagnostic value in distinguishing between patients with neurodegenerative disorders and healthy controls 131. Certain salivary redox biomarkers are very sensitive to age, comorbidities, and medication, which increases their usefulness as diagnostic and prognostic markers. In general, saliva-derived EVs are a valuable resource for non-invasive liquid biopsy, as they represent the physiological and pathological conditions of their parent cells from systemic circulation. Analysis of proteins and nucleic acids inside salivary EVs is essential to biomarker discovery. However, it can be challenging to isolate and characterize salivary EVs due to their low abundance and the complexity of saliva, resulting in variable detection results. Standardizing the procedures and maximizing the quantity and quality of EVs in saliva will require further studies. It is promising to have the nucleic acid biomarkers together with the protein biomarkers and metabolites to provide a precise diagnosis in saliva-derived EVs.

## Saliva collection methods

Natural saliva and stimulated saliva have distinct differences in their production, composition, and function. Natural saliva, mainly secreted from the sublingual and submaxillary glands, is produced at rest without any external stimuli. It has a flow rate of 0.3-0.7 mL/min and a pH range of 6.5-7.5, contains a baseline concentration of electrolytes, enzymes, and antimicrobial agents, and plays a critical role in protecting oral and peri-oral tissues, providing lubrication, buffering capacity, remineralizing teeth, restoring soft tissues, and aiding digestion. In contrast, stimulated saliva is produced in response to various stimuli, such as taste (gustatory) or mechanical actions (like chewing), 80% of stimulated saliva is secreted by the parotid gland. It often contains high concentrations of bicarbonates, which act as buffers, and other components that aid in digestion and oral health. The flow rate of stimulated saliva ranges from 1.5 to 2 mL/min, with a lower pH range of 6.0-7.5. In short, the key differences between natural and stimulated saliva lie in their flow rates, composition, and functional roles in the oral cavity, helping with dental health assessment and management of saliva function-related diseases.

**Figure 2:**
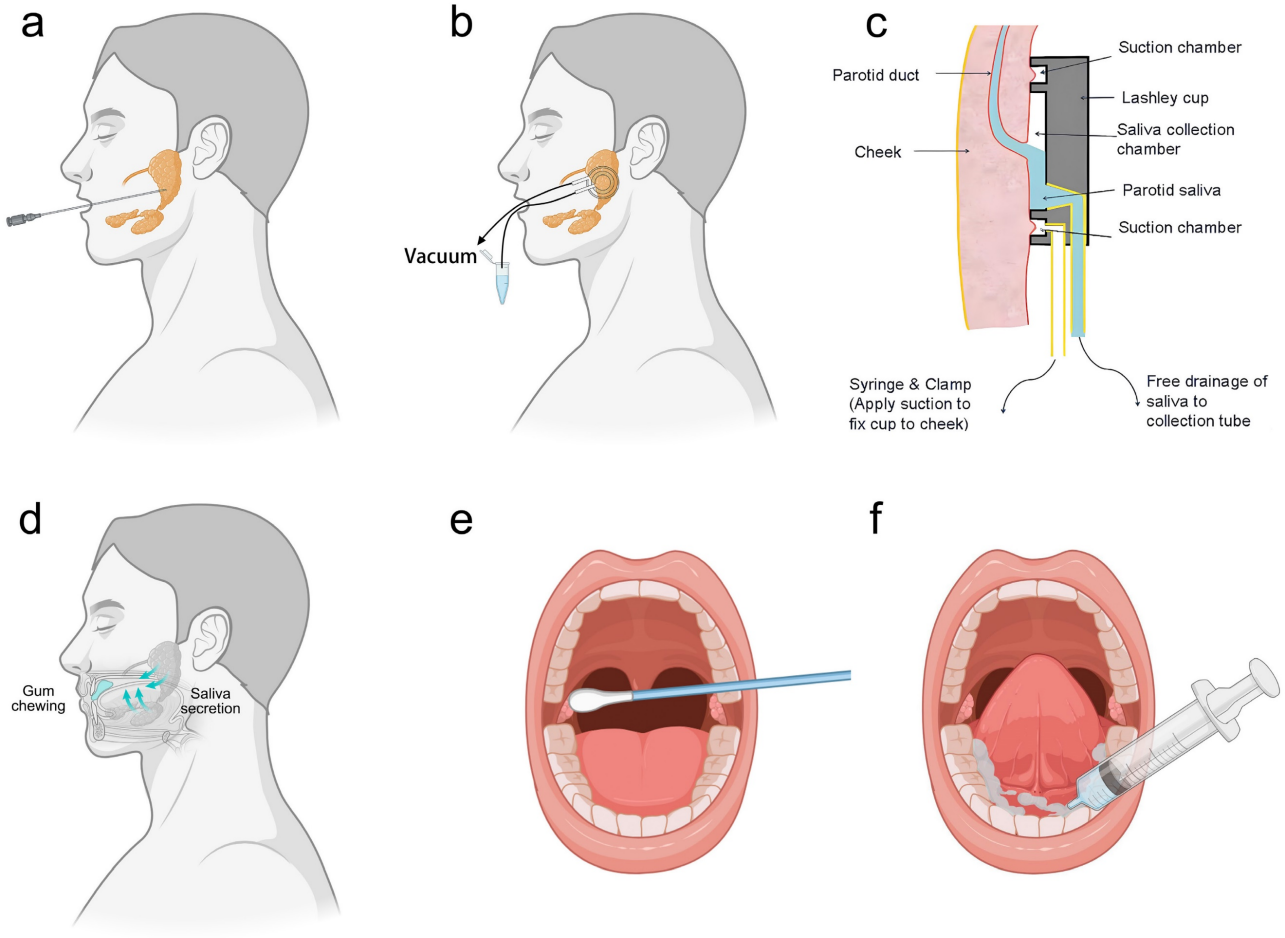
Saliva sampling methods. (a) Stimulated collection of saliva from the parotid gland using sialographic cannulas. (b) Stimulated/unstimulated collection of saliva from parotid gland using Lashley cup. (c) Lashley cup structure. (d) Stimulated saliva produced by chewing gum. (e) Unstimulated collection of saliva from the parotid gland. (f) Unstimulated submandibular and sublingual saliva collection.

Stimulated saliva collection involves the physical or chemical stimulation of the oral cavity to produce saliva, which can be collected from specific glands, such as the parotid glands, or as whole saliva. For instance, parotid gland saliva is preferred for clinical diagnosis due to its lower contamination levels but requires long sampling times, professional staff, and specialized equipment like polyethylene tubes or sialographic cannulas (**Fig. 2a**). Moreover, less invasive methods include the Carlsson-Critten collector or Lashley cup, which use citric acid stimulation and metallic tubes to collect saliva (**Fig. 2b, 2c**). On the other hand, whole saliva is stimulated through activities like chewing, which can significantly alter its composition and flow rate (**Fig. 2d**).

In contrast, natural saliva is preferred in clinical and laboratory research because it minimizes analyte dilution. However, collecting natural saliva also needs standardized procedures. For example, unstimulated parotid saliva can be collected using Lashley cups or cotton swabs, though the latter may require longer collection times for adequate volume (**Fig. 2e**). Additionally, submandibular and sublingual saliva can be systematically collected from the oral cavity floor using syringes and Lashley cups (**Fig. 2f**). Regardless of the collection method, it is essential to minimize contamination with food residues or other substances that may affect test results. By understanding the differences and appropriate collection methods for natural and stimulated saliva, more accurate and reliable diagnostic and research outcomes. **Table 2** provides the comparison of stimulated saliva collection and unstimulated saliva collection, guiding the selection of saliva collection methods.

## Post-collection processing of saliva

**Figure 3:**
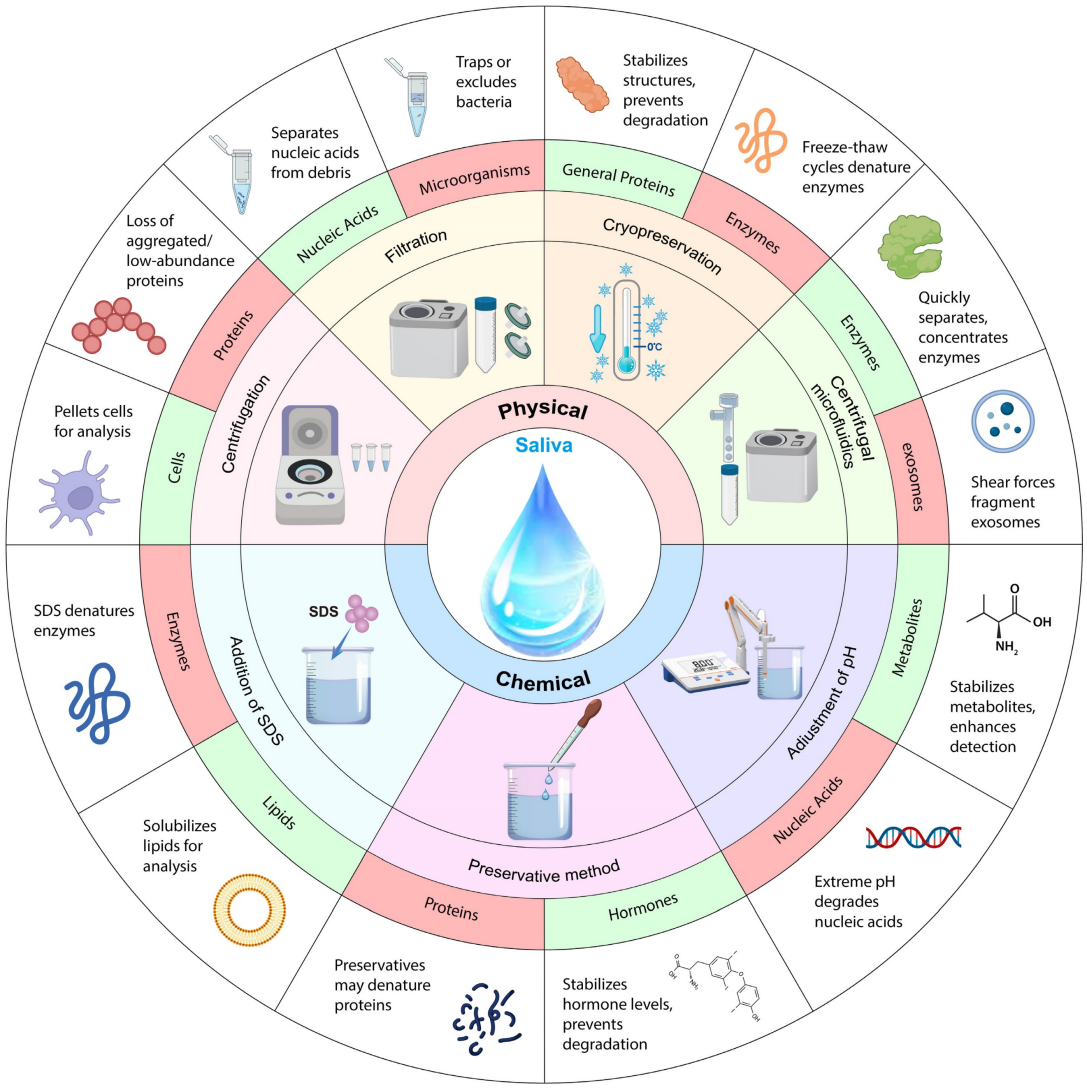
Schematic diagram of saliva processing. Among different saliva processing methods, green indicates processing applicable objects; red indicates processing inapplicable objects.

Saliva processing is a vital step in ensuring the accuracy and reliability of diagnostic tests. After saliva collection, saliva samples may undergo centrifugation or filtration to remove particulates and cells to isolate specific components such as hormones, enzymes, or antibodies. Additionally, some tests might necessitate the addition of preservatives or chemicals to stabilize the sample and preserve essential biomarkers. To maintain the integrity of the sample throughout storage and transit, buffer or pH adjustments need to be made during saliva processing. The different saliva processing methods, their advantages and disadvantages, and detection techniques are listed in **Table 3**. Normally post-saliva collection processing includes physical methods (centrifugation 141, filtration 142, cryopreservation 143, and centrifugal microfluidics 144) and chemical methods (addition of sodium dodecyl sulfate (SDS) 145, adjustment of pH 146, and preservation with preservatives 147) as shown in **Fig. 3**.

## Saliva detection methods

### Methods beyond biosensors

Polymerase chain reaction (PCR) and real time PCR are commonly used for detecting nucleic acids, including DNA and RNA, in saliva. Kang *et al.* utilized real-time quantitative PCR and enzyme-linked immunosorbent assay (ELISA) to determine the expression of miR-23a, miR-146a, IL-1β, IL-6, and IL-17 in the saliva of patients and healthy volunteers, which can be used to diagnose periodontitis 148. In the context of COVID-19, saliva has proven to be a reliable specimen for detecting SARS-CoV-2 using RT-PCR 149. Additionally, saliva samples have been used to assess differential gene expression of immune response molecules and cellular enzymes in patients with mild, moderate, and severe COVID-19 150. However, contamination during PCR can result in misleading positive results 151. To realize the high-throughput analysis, microarray technology has been used to study gene expression in saliva to identify biomarkers for oral feeding readiness in preterm infants and assess the levels of pro-inflammatory cytokines in patients treated with fixed orthodontic appliances 152,153.

In addition to PCR and ELISA, other methods such as mass spectrometry (MS), Raman spectroscopy, electric field-induced release and measurement (EFIRM) and next-generation sequencing (NGS) are popular in saliva detection. They are capable to identify and analyze various biomarkers in saliva, including those related to transcriptomics, proteomics, genomics, and metabolomics.

As a highly sensitive, universal technique in analytical science, MS allows for the detection of several oral and systemic disorders and the prediction of their course, as well as the determination of the biological profile under diseased and normal settings. For proteome investigation, liquid chromatography-mass spectrometry (LC-MS) is thought to be very sensitive and adaptable, however, it has limits when it comes to finding peptide traces 154. This limitation is the low ionization efficiency (by protonation or deprotonation) and the low ion stability in the MS experiment. Furthermore, quantitative analysis by LC-MS frequently cannot be performed without isotopically labeled standards, which usually have to be specially synthesized. Gas Chromatography-Mass Spectrometry (GC/MS) is used to evaluate plasticizer exposure in dentistry. This technique involves analyzing saliva samples after hollow fiber liquid phase microextraction to identify the metabolites of phthalates and bisphenol A, which are widely used in dental materials 155. As GC-MS is utilized for the analysis of volatile and semi-volatile compounds, large molecules or high molecular weight compounds in saliva are not suitable for GC-MS detection.

Raman spectroscopy offers non-destructive quantitative analysis of the chemical composition and structure of analytes, with an extremely low detection limit of 100 ppb. Currently, microfluidic technology has been combined with Raman spectroscopy to simultaneously monitor multiple analytes in a single microfluidic channel. This combination makes microfluidic Raman spectroscopy applicable for reaction monitoring, fiber probes, and identification of tumor cells 156,157,158.

EFIRM is an innovative liquid biopsy platform designed to detect circulating tumor DNA (ctDNA) containing specific mutations directly from bodily fluids like saliva and plasma. EFIRM utilizes an electric field to preferentially release and concentrate single-stranded DNA molecules, including ultra-short ctDNA fragments. Its key advantages include high sensitivity, with the ability to detect single-digit copy numbers of mutations, a rapid 3-hour assay turnaround time, cost-effectiveness compared to NGS, and minimal sample volume requirements (30 μL). EFIRM has demonstrated high sensitivity in detecting actionable mutations, such as EGFR mutations in non-small cell lung cancer (NSCLC), from saliva and plasma samples. It supports both qualitative (mutation detection) and quantitative (mutation load monitoring) assays, with potential for multiplexing and point-of-care applications, exemplified by a single-droplet microsensor for multiplexed EGFR mutation detection. However, EFIRM is currently limited to detecting a few specific mutations, mainly in EGFR for NSCLC, and has limited multiplexing capabilities in its present hardware configuration. Its sensitivity might be affected by the prevalence of targeted mutations in different populations, and further validation and standardization are necessary for clinical implementation 159.

NGS refers to modern sequencing techniques that can rapidly sequence millions or billions of DNA molecules in parallel. Illumina (sequencing by synthesis), Ion Torrent (semiconductor sequencing), and Oxford Nanopore (nanopore sequencing) are popular technologies for massively parallel sequencing, producing vast amounts of data quickly and cost-effectively compared to the older Sanger sequencing method. NGS facilitates the discovery of novel genes and variants with applications in research and clinical settings. However, NGS also has limitations, including shorter read lengths, higher error rates, significant computational demands, potential technical biases, and ethical and privacy concerns related to large-scale genomic data generation 160.

### *In vitro* biosensors

Biosensors are analytical devices that combine a biological component, such as enzymes, antibodies, or nucleic acids, with a physicochemical detector to recognize and measure analytes. Current biosensing devices have shown advantages over traditional detection technologies in screening and diagnosing highly recurrent diseases, such as low cost, short diagnosis time, and real-time monitoring. They are particularly useful in salivary detection due to their ability to provide rapid, specific, and sensitive analysis of various biomarkers, such as nucleic acids, proteins, small molecules, or ions, making them versatile tools in both clinical diagnosis and research 161.

**Figure 4:**
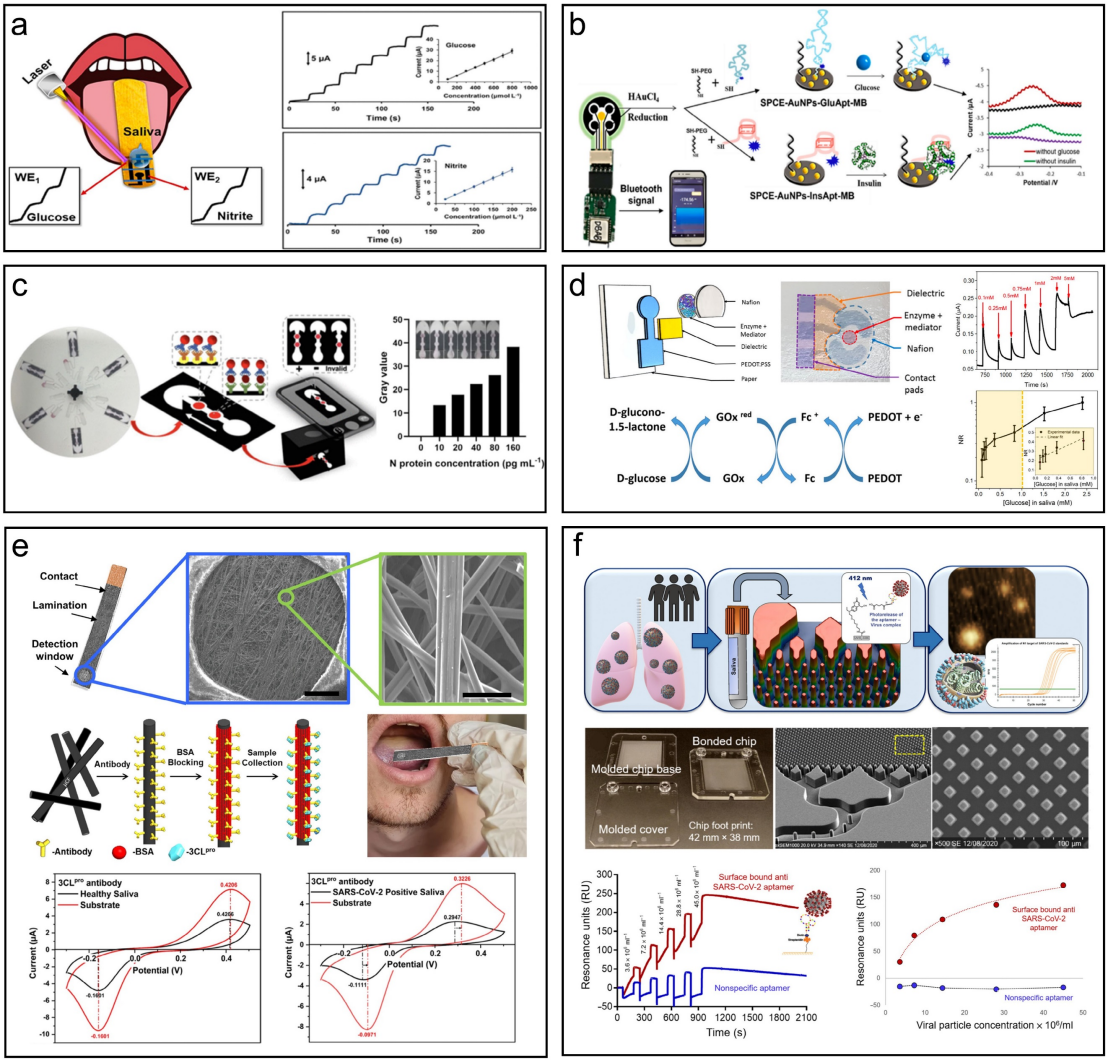
Summary of *in vitro* saliva detection devices. (**a**) A laser-treated wooden tongue depressor is transformed into an electrochemical multiplex biosensing device for oral fluid analysis. Reproduced with permission 162. Copyright 2023, American Chemical Society. (b) An electrochemical aptasensor for simultaneous detection of glucose and insulin on a portable biochip with smartphone signal readout. Reproduced with permission 163. Copyright 2022, ELSEVIER. (c) μPADs on centrifugal microfluidic discs for the quantification of the N protein of pseudovirus in saliva. Reproduced with permission 144. Copyright 2022, American Chemical Society. (d) A disposable paper glucose sensor printed with conductive polymer, uses a glucose oxidase enzyme and electron mediator for biorecognition. Reproduced with permission 164. Copyright 2018, Nature. (e) SARS-CoV-2 infection detection in saliva using microfluidic electrochemical analysis of 3CLpro enzymatic activity. Reproduced with permission 167. Copyright 2022, Nature. (f) A microfluidic chip detects active SARS-CoV-2 virus from saliva for rapid and accurate infection identifications. Reproduced with permission 168. Copyright 2022, Science.

Electrochemical biosensors present a highly promising approach for achieving highly sensitive, selective, multiplexed, and cost-effective detection of proteins and nucleic acids *in vitro*. Koukouviti *et al.* designed a wooden tongue depressor (WTD) multiplex biosensor for the simultaneous determination of glucose and nitrite in artificial saliva (**Fig. 4a**) 162. The WTD was transformed into an electrochemical multiplex biosensing device for oral fluid measurement by laser treatment. The WTD's surface was programmably irradiated by an inexpensive laser engraver fitted with a low-power (0.5 W) diode laser, creating two miniature electrochemical cells (e-cells). Using a commercial hydrophobic marker pen, programmed pen-plotting is used to spatially divide the two e-cells. The limits of detection were 22.5 and 3.5 μmol/L for glucose and nitrite. Liu *et al.* created an electrochemical aptasensor on a screen-printed carbon electrode (SPCE) for non-invasive simultaneous real-time detection of glucose and insulin in saliva (**Fig. 4b**) 163. The sensing interface responded linearly to glucose in the range of 0.1-50 mM with a detection limit of 0.08 mM and to insulin in the range of 0.05-15 nM with a detection limit of 0.85 nM when using the electrochemical signal readout on SPCE. By combining a portable wireless biochip with an SPCE-based sensing interface, it was possible to provide continuous real-time, non-invasive glucose and insulin monitoring in saliva using smartphone signal reading. They also generated a microfluidic paper-based analytical device (μPAD) to detect SARS-CoV-2 Nucleocapsid (N) protein in saliva with high specificity (**Fig. 4c**) 144. With a dynamic range of 10-1000 pg/mL and an assay period of 8 min, the on-disc μPAD was able to detect the SARS-CoV-2 N protein down to 10 pg mL^-1^ by chemically treating the μPAD surface and adjusting the protein immobilization conditions. Bihar *et al.* developed an electrochemical glucose sensor on paper substrates for non-invasive glucose monitoring utilizing inkjet-printing technology (**Fig. 4d**) 164. The design incorporates a conducting polymer (PEDOT: PSS) and glucose oxidase as the biorecognition element.

This sensor detects a broad range of glucose concentrations in saliva and retains functionality with minimal performance loss (<25%) up to a month after fabrication when sensors were stored in air-free conditions. Similarly, a highly sensitive glucose sensor was developed on a conductive nanomesh made of linked metal-organic frameworks (MOF) with a detection limit of 16.57 µm, which remains stable for 16 days of usage 165. Escuela *et al.* summarized the challenges faced by enzymatic detection, which is more stringent for conditions such as pH, temperature, and ion strength 166. A rapid electrochemical detection system for SARS-CoV-2 infection was developed, harnessing 3CLpro enzymatic activity in unprocessed saliva (**Fig. 4f**) 167. This cutting-edge design employs electrochemical biosensing to identify viral protease activity in saliva within one minute, providing swift, point-of-care diagnostics for active infections. The system incorporates a conductive carbon paper electrode (CPE) functionalized with 3CLpro-specific antibodies to capture the viral enzyme, triggering a redox reaction measurable via cyclic voltammetry (CV). The pH-responsive quinone probe, p-benzoquinone, exhibits a shift in redox potential in response to enzymatic activity, delivering a highly sensitive and specific signal. Clinical trials with saliva samples from 50 individuals revealed 100% sensitivity and specificity, validating the method's effectiveness for large-scale screening without the need for sample preparation or specialized equipment. This approach reduces the dependency on costly reagents and offers a rapid, user-friendly solution for SARS-CoV-2 detection.

RT-qPCR and rapid antigen testing have remained the cornerstone of COVID-19 detection, but they fail to distinguish between patients with active, potentially infectious disease and those who lack the ability to transmit the virus. As a result, PCR-based tests may overestimate the number of active disease spreaders. To address this issue, Gamage *et al.* devised a microfluidic chip capable of isolating and detecting active SARS-CoV-2 particles from saliva, specifically targeting viral particles with an accessible receptor-binding domain (RBD) on the spike protein. Utilizing surface-bound DNA aptamers for affinity selection, intact virus particles are captured by the chip's microstructures and subsequently released via blue light for RT-qPCR detection (**Fig. 4e**) 168. The system exhibits remarkable specificity, effectively distinguishing between active and non-active viruses—critical for identifying truly infectious individuals. Boasting a recovery rate of approximately 94%, this device holds promise for scalable, low-cost production, making it well-suited for point-of-care testing. Moreover, it offers a simple, affordable method of extended quarantines by more accurately determining infectiousness than conventional RT-qPCR, which often detects inactive viral RNA.

### Oral wearable biosensors

Oral wearable monitoring is used in the oral environment to monitor the oral health status or treatment process continuously 169,170. It normally consists of a sensor module, a waterproof housing, a data processing unit, a communication module, an energy module, a fixture, and additional components like LED indicators and push-button switches. Together, these components form the basic framework of the wearable sensor, enabling effective monitoring and management of oral health conditions and treatment processes. Being different from *in vitro* detection, all materials used in oral wearables should be biocompatible 171,172.

**Figure 5:**
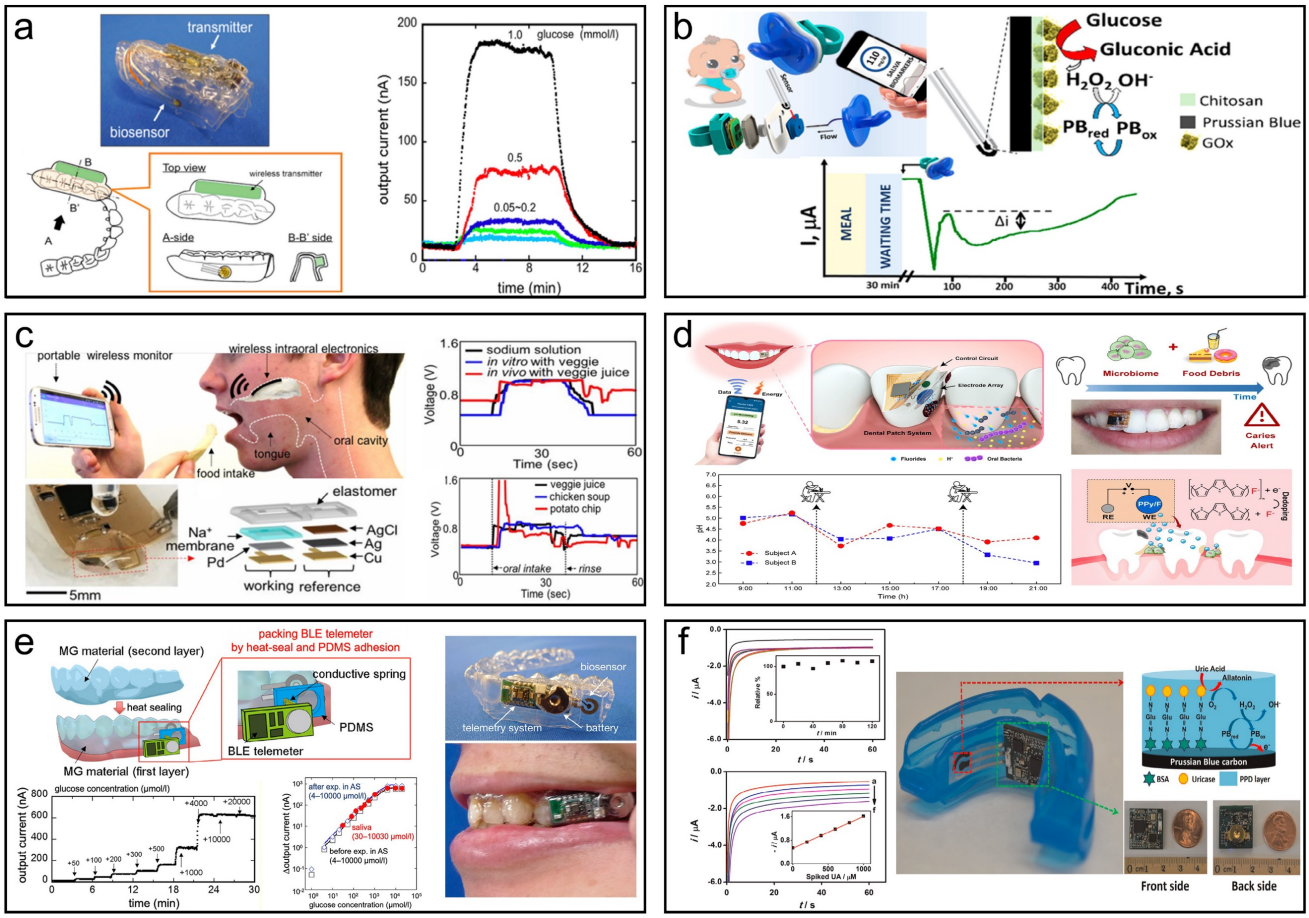
Summary of oral wearable monitoring devices. (a) Mouthguard glucose sensor and its reaction with the open-loop injection system and phantom jaw. Reproduced with permission 173. Copyright 2016, ELSEVIER. (b) Pacifier Biosensor and schematic amperometry response of glucose monitoring. Reproduced with permission 174. Copyright 2019, American Chemical Society. (c) The intraoral sodium intake sensor enables real-time responses to various diets *in vivo* by a human subject. Reproduced with permission 175. Copyright 2018, PNAS. (d) Monitoring fluctuations in the local oral microenvironment pH using wearable devices to detect caries lesions Reproduced with permission^176^. Copyright 2022, Nature. (e) A wearable cellulose acetate-coated mouthguard biosensor for *in vivo* salivary glucose measurement. Reproduced with permission 177. Copyright 2020, American Chemical Society. (f) A mouthguard biosensor for uric acid that has built-in wireless electronics. Reproduced with permission 178. Copyright 2015, ELSEVIER.

Nowadays, more and more wearable saliva biosensors that integrate sampling, diagnosis, and signal output have emerged, providing convenience for real-time monitoring of disease. Takahiro *et al.* presented a mouthguard biosensor for monitoring glucose within 5-1000 µmol/L in human saliva 173. The wearable sensor has a wireless communication module's capacity to track salivary glucose levels in a prosthetic jaw that closely resembles a person's mouth. Over a period of 5 h, the telemetry system allows for consistent, long-term real-time, non-invasive salivary glucose monitoring (**Fig. 5a**). García-Carmona *et al.* described the first chemically wearable sensor for neonates, obtaining limit of detection (LOD) and quantification (LOQ) of 0.04 mM and 0.1 mM, respectively, which are sufficient to quantify routine blood glucose levels in diabetic patients and successfully monitor glucose levels in infants, opening up new possibilities for the use of saliva as a non-invasive sample for monitoring infants' and neonates' internal metabolites (**Fig. 5b**) 174. Lee *et al.* developed an intraoral biosensor to measure sodium in food during food intake, with a sensitivity that clearly detects concentration differences as well as high sensitivity (as small as 10^-4^ M solution), providing an adequate sensing spectrum for sodium detection, which could be used to control hypertension (**Fig. 5c**) 175. Liu *et al.* have developed a wearable, battery-free dental patch designed for real-time monitoring of the oral microenvironment. The system integrates near-field communication (NFC) technology with an electrochemical sensor, utilizing pH-sensitive electrodes to detect localized acidity changes caused by microbial metabolism. The sensor exhibits high sensitivity and selectivity, accurately monitoring pH fluctuations in the oral cavity and issuing alerts via smartphone when potential caries are detected. Both *in vitro* and *in vivo* experiments demonstrate the system's precision, offering an effective method for early detection of dental caries 176. Takahiro *et al.* developed a mouthguard glucose sensor with a cellulose acetate interference suppression membrane, addressing the challenge of accurately detecting salivary glucose, influenced by ascorbic and uric acids. It measures glucose concentrations in saliva efficiently without pre-treatment, in the range of 1.75 to 10,000 μmol/L, making it appropriate for tracking average salivary glucose levels (20-200 μmol/L). The accuracy of the sensor was verified by testing using saliva samples from healthy (**Fig. 5e**) 177. Wang *et al.* also developed a mouthguard with instruments that can measure salivary UA levels non-invasively. SPCE modified by uricase has been integrated onto a mouthguard platform, accompanied by anatomically minimized instrumentation electronics that include a microprocessor, potentiostat, and Bluetooth low energy transmitter. The new platform allows real-time wireless transmission of the sensed data to common cellphones, laptops, and other consumer electronics for on-demand processing, diagnostics, or storage, in contrast to RFID-based biosensing systems, which necessitates huge proximal power supplies. The mouthguard biosensor covers the concentration ranges for both healthy individuals and patients with hyperuricemia (0-1 mM UA) and offers excellent sensitivity (2.45 μA/mM), selectivity, and stability for salivary UA detection. The novel wireless mouthguard biosensor created an appealing wearable system for real-time monitoring of a range of fitness and health applications (**Fig. 5f**) 178. These technologies and advancements in salivary diagnostics hold great potential for early detection, monitoring, and management of various diseases, offering a more accessible and less invasive alternative to traditional diagnostic methods.

Both *in vitro* biosensors and wearable devices for saliva detection demonstrate substantial diagnostic value across a spectrum of clinical scenarios. *In vitro* methods are particularly suited for routine screenings, disease monitoring, and the rapid diagnosis of infectious diseases such as COVID-19 and HIV, alongside the assessment of endocrine disorders like diabetes and Cushing's syndrome, and the detection of biomarkers for various cancers and dental diseases. Large sample numbers, standardized data analysis techniques, high sensitivity, and specialized sensors are the main requirements for *in vitro* saliva detection. In contrast, wearable devices enable continuous, personalized, and remote health monitoring, proving essential for the management of chronic diseases such as diabetes and hypertension, the evaluation of hydration and electrolyte balance in athletes, and the tracking of stress-related biomarkers for mental health. The need for wearables that are easy to use and comfortable, sensors with high sensitivity and specificity, device self-calibration, and algorithms that can correctly interpret sensor data are frequent challenges associated with oral wearable monitoring.

## Challenges of and solutions to salivary diagnosis

Saliva, as a non-invasive and easy-to-collect biological sample, has shown great success in the screening of COVID-19 infection during the global epidemic. However, there are still many challenges in using saliva as an alternative body fluid in diagnosing diseases clinically, such as saliva sample collection/storage, ingredient complexity, sensitivity, and standardization of saliva diagnosis.

### Collection of saliva samples

The quality of salivary samples, crucial for reliable and accurate diagnostics or research outcomes, is affected by numerous factors. One key factor is the collection method, which includes passive drool, swab, or stimulated collection, which may impact the sample's composition. Research shows stimulation methods may dilute specific biomarkers like Na^+^ and K^+^ 179. The timing of collection is equally important as saliva composition varies throughout the day due to circadian rhythms. Numerous enzymes, hormones, and active molecules are present in saliva, and its amount and content are modified within regular daily intervals, making the timing of collection an important consideration for accurate analysis 141. Research has shown that salivary flow rate and the concentrations of various salivary substances, such as protein, sodium, potassium, calcium, and chloride, follow circadian rhythms.^180^. Therefore, it is recommended to collect saliva samples at specific times of the day to account for these variations. In addition, attention should also be paid to food and drink intake during the saliva collection process. Since different diets might affect the makeup of saliva. For example, salivary pH levels typically rise right after eating, and food intake can affect salivary flow rate. In addition, it has been found that eating before saliva collection can affect the recognition of viral nucleic acids in saliva. Therefore, it is advisable to avoid consuming food as much as possible to reduce the impact on saliva composition. 181.

### Storage and stability of saliva samples

The storage temperature of saliva samples greatly affects their stability. To guarantee the integrity of the analytes being tested, saliva samples must be stored and kept stable. For short-term storage, several saliva analytes are more stable at 4 ºC than at room temperature, but for long-term storage, they are generally more stable at -20 ºC or -80 ºC 182. Enzymes (amylase and protease) in saliva may degrade certain biological molecules. Therefore, preservatives such as protease inhibitors, RNase inhibitors, and DNA/RNA stable solutions need to be added to maintain sample stability. Saliva samples that have DNA stability can be kept for up to 18 months at 37 ºC without affecting their quality or capacity to withstand different methods 183. Dried saliva spot (DSS) is an innovative sampling technique that involves collecting saliva samples on filter paper, which are then allowed to dry. Saliva remains stable in DSS for up to a month at various temperatures, making it a popular alternative to serum for detecting target molecules. Krone *et al.* described a reliable method for detecting the presence of Streptococcus pneumoniae in human saliva using polymerase chain reaction and the WhatmanTM 903 protein saver card 184. In addition, if there are bacteria, viruses and other microorganisms in saliva, the sample will be contaminated and the analysis interfered, so sterile equipment and consumables should be used. At the same time, the collection method and targeted analytics may also affect the stability of saliva samples. When collecting and storing saliva samples, researchers and doctors should carefully consider these parameters to ensure the accuracy and reliability of follow-up studies.

### Complexity of salivary components

Saliva contains various components, such as enzymes, nucleic acids, hormones, cells, microorganisms, etc. The salivary composition is influenced by various factors such as age, gender, diet, and cleaning products, therefore it has high individual differences 134. This poses significant difficulties in obtaining standardized saliva samples. In addition, the above factors may also reduce the accuracy and reliability of the results. Addressing the challenge of variability in salivary composition for clinical diagnostics necessitates a multifaceted approach, centering on the establishment of strict collection protocols that encompass guidelines on patient preparation, such as fasting, maintaining oral hygiene, and standardizing the time and method of saliva collection 141. Implementation of consistent pre-analytical treatments like centrifugation and the use of specific preservatives to maintain biomarker stability is equally crucial. When interpreting results in the context of individual variations, it is essential to gather thorough patient data, including age, gender, food, medication, and lifestyle factors. By using sophisticated analytical methods like mass spectrometry or high-performance liquid chromatography, salivary component detection and quantification are strengthened, which reduces the effect of sample variability 185. Additionally, standardization methods, such as adjusting for total protein content or saliva volume, can further mitigate variability effects 186. Hartl *et al.* utilized an integration of statistical modeling and machine learning algorithms to decipher complex datasets and adapt to inherent variability 187. In addition, regular quality control and equipment calibration are required to maintain analytical accuracy. Collaborating with other laboratories and institutions to develop and adhere to standardized protocols and best practices can significantly improve the reliability and comparability of saliva diagnostic results in different environments. By weaving together these strategies, the challenges posed by the complexity and variability of salivary components can be effectively mitigated, enhancing the reliability and clinical utility of salivary diagnostics.

### Detection sensitivity

In terms of sample processing, sample concentration techniques such as ultrafiltration or freeze-drying can improve the detectable level of biomarkers. Cutting edge detection technologies such as ultra-high performance liquid chromatography and tandem mass spectrometry can amplify signals by improving their sensitivity and separation efficiency. Nucleic acid amplification technology is also changing the biological testing industry, providing more opportunities for saliva diagnosis while maintaining high efficiency, specificity, and cost-effectiveness 188,189. Nanomaterials such as carbon nanotubes, graphene, and metal nanoparticles are being used to develop highly sensitive and selective electrochemical biosensors for saliva biomarkers. These nano material-based biosensors can detect various molecules in saliva, including glucose, hormones, proteins, viruses, and bacteria 190,191. Under the premise of standardized sampling, precise data analysis techniques and improved algorithms also contribute to improving the sensitivity of detection 187. In addition, measuring multiple indicators simultaneously can greatly improve the overall sensitivity and specificity of saliva detection. By integrating these advanced scientific methods, saliva testing is expected to become a more effective tool for non-invasive diagnosis and early detection in clinical settings.

### Standardization of salivary diagnosis

Salivary diagnostics must be standardized to ensure that the results of various studies and laboratories are reliable, reproducible, and comparable. Its main purpose is to develop unified methods, processes, and quality control measures for collecting, processing, and examining saliva samples to minimize individual differences as much as possible. There are commercial saliva testing products on the market today that use a variety of standardized methods to ensure the accuracy and reliability of test results. Salimetrics is one such entity, offering a range of products and services for saliva collection and testing. They emphasize the importance of proper saliva collection and processing procedures to obtain high-quality, reproducible data. This includes understanding the variability of saliva composition and being mindful of factors such as time of collection, oral position of the collection swab, and salivary flow rate. They also advised against the use of oral stimulants during collection to minimize unnecessary variability in test results 185. Creating accurate and standardized analytical instruments, establishing reference intervals, and carrying out round-robin trials are all part of achieving standardization.

## Conclusion

In this review, we introduce relevant diseases from the perspective of biomarkers in saliva and focus on the latest technologies and challenges in saliva diagnosis. Ongoing research on salivary diagnosis may uncover new salivary biomarkers for the diagnosis and monitoring of various diseases. In a variety of clinical settings, patients and healthcare professionals find it to be an appealing option due to its non-invasive nature and ease of collection. Saliva collection greatly reduces the risk of infection during sampling for both healthcare professionals and patients. The non-invasive saliva collection effectively blocks the spread of harmful pathogens such as HIV and hepatitis virus and eliminates the risk of infection caused by improper disinfection, which is of great benefit to some countries with poor medical resources and medical standards. In addition, for patients who are sensitive to pain and have blood sickness, saliva sampling can greatly improve their compliance. Saliva testing is a safer and easier alternative to traditional biological fluid (blood and urine) screening, which can be used for rapid testing of large populations to enhance infectious disease detection and monitoring. With the application of new technologies such as biosensing and microfluidics in saliva diagnosis platforms, the development trend of saliva detection equipment has shifted towards higher sensitivity, faster reaction time, smaller device size, portability, and automation to meet the needs of different fields, including disease diagnosis and management, drug screening and personalized medicine. However, the stability and standardization issues in the pre-treatment process of saliva samples still pose significant challenges to the accuracy of detection results. Currently, numerous salivary biomarkers lack standardized reference ranges and individual variability among subjects further complicates sample standardization. To address these challenges, researchers and clinical practitioners should implement rigorous laboratory and analytical methods and actively pursue research on standardization and method improvement. This effort aims to enhance the reliability and accuracy of saliva sample analysis. The potential for saliva analysis in non-invasive disease diagnosis, management, and prevention is vast and promising.

## Figures and Tables

**Table 1:** Major components in saliva and their relevance with various diseases.

Salivary components		Concentration in saliva	Concentration in blood	Biological significance	Related diseases	Detection methods
Proteins and peptides	Amylase	0-7.5 mg/mL 26	10-110 µg/mL	Aids digestion, protects the oral mucosa improves the taste of food	Pancreatitis, sialadenitis, child development, malnutrition	Enzyme-linked immunosorbent assay (ELISA), mass spectrometry (MS), polymerase chain reaction (PCR)
	Prostate-Specific Antigen	<4.0 ng/mL 27	N/A	Monitor for prostate-related disease	Prostate cancer, prostate cancer	Enzyme-linked immunosorbent assay (ELISA), Mass spectrometry (MS), molecular diagnostic techniques
	Lysozyme	5.9-17.3 μg/mL 28	7-13 µg/mL 29	Antibacterial, aids digestion, promotes wound healing	Periodontal disease and dental caries, enteritis, rheumatoid	Enzyme-linked immunosorbent assay (ELISA), western blotting (WB)
	Glutathione peroxidase	7.2-165.2 mg/mL 30	263-639 mg/mL 31	Anti-bacterial, anti-inflammatory, promotes wound healing	Periodontal disease gingivitis, diseases of salivary gland function, digestive system diseases	Enzyme-linked immunosorbent assay (ELISA), mass spectrometry (MS), western blotting (WB), chromatography, biosensors, flow cytometry
	Lipase	13 µg/mL 32	0-0.16 U/mL 33	Promote fat digestion, improve food taste, participate in immune response	Dry mouth, gastroesophageal reflux disease, diabetes mellitus	Enzyme-linked immunosorbent assay (ELISA), colloidal gold method, liquid chromatography-tandem mass spectrometry (LC-MS), spectrometry
	sIgA	4-10.3 µg/mL 34	N/A	Protect local immune, regulate immune responses, clear pathogens, maintain oral microbial stability, promote wound healing	Crohn's disease, ulcerative colitis, multiple myeloma	Enzyme-linked immunosorbent assay (ELISA), western blotting (WB), immunodiffusion assay, immunoturbidimetry assay
	IgG	0.05-0.36 mg/mL 35	0.4-0.7 mg/mL 35	Promote fat digestion, improve food taste, and participate in immune response	Dry mouth, gastroesophageal reflux disease, diabetes mellitus	Enzyme-linked immunosorbent assay (ELISA), fluorescence method, lateral flow assay, liquid chromatography-tandem mass spectrometry (LC-MS), spectrometry
	IgM	N/A	0.4-2.3 mg/mL 36	Respond to pathogen intrusion	Rheumatoid arthritis, systemic lupus erythematosus, oral cancer, HIV	Enzyme-linked immunosorbent assay (ELISA), mass spectrometry (MS), western blotting (WB)
	MMP8	1.85-31.65 ng/mL 37	0.97-6.17 ng/mL 38	Remodel oral tissue, inflammatory response, as a diagnostic marker for oral and systemic diseases	Oral cancer, rheumatoid arthritis, sjögren's syndrome	Enzyme-linked immunosorbent assay (ELISA), mass spectrometry (MS), western blotting (WB), polymerase chain reaction (PCR) immunofluorescence (IF) staining
	MMP9	0.1-1.5 ng/mL 39	0.5-5 ng/mL 40	Remodel oral tissue. inflammatory response, as a diagnostic marker for oral and systemic diseases	Oral squamous cell carcinoma, oral potentially malignant disorders	Enzyme-linked immunosorbent assay (ELISA), mass spectrometry (MS), western blotting (WB), polymerase chain reaction (PCR) immunofluorescence (IF) staining
	Mucins	15-240 mg/mL 41	9.1- 10.7 ng/mL 42	Lubricate, maintain the stability of oral microorganisms, regulate the immune response, and improve the taste of food	Xerostomia, oral mucositis, reflux esophagitis, gastric ulcer, chronic bronchitis, asthma, Sjogren syndrome, systemic lupus erythematosus, diabetes, obesity	High-performance liquid chromatography (HPLC), mass spectrometry (MS), western blotting (WB), real-time polymerase chain reaction (RT-PCR) 43, immunofluorescence (IF) staining
	Proline-rich proteins	2-4 mg/mL 44	N/A	Antimicrobial action, mineral deposition, and dental plaque formation improve food taste	Dental caries, periodontal disease, oral cancer	Enzyme-linked immunosorbent assay (ELISA), liquid chromatography-tandem mass spectrometry (LC-MS), nuclear magnetic resonance spectroscopy (NMR), real-time polymerase chain reaction (RT-PCR), 2D gel electrophoresis (2-DE)
	Albumin	0.1-0.8 mg/mL 45	34-54 mg/mL 46	Lubricate, maintain osmotic pressure, promote wound healing, and transport drug hormone molecules	Gastroesophageal reflux disease, malnutrition	Enzyme-linked immunosorbent assay (ELISA), mass spectrometry (MS), western blotting (WB), immunodiffusion
	Transferrin	2-21.2 µg/mL 47	2-3.6 mg/mL 48	Transport regulation of iron, antibacterial action, immunomodulation	Iron anemia, chronic kidney disease, malnutrition	Enzyme-linked immunosorbent assay (ELISA), nuclear magnetic resonance spectroscopy (NMR), mass spectrometry (MS),
	Growth Hormone	N/A	<10 ng/mL 49	Monitor growth hormone levels	Renal insufficiency, acromegaly, turner syndrome, Prader-Willi syndrome	Enzyme-linked immunosorbent assay (ELISA), polymerase chain reaction (PCR)
Nucleic acids	miRNA	2.59-29.4 μg/mL 50	4.4-10.9 pM 51	Biomarkers, immune regulation, affect cell proliferation and apoptosis, regulate gene expression	Cancer, coronary heart disease, myocardial infarction, Alzheimer's disease, Parkinson's disease, multiple sclerosis	Real-time polymerase chain reaction (RT-PCR), RNA sequencing (RNA-Seq), nanostring technology, DNA microarray technology
	circRNA	N/A	N/A	Biomarkers, regulate gene expression and participate in local immune responses	Cancer, neurodegenerative diseases	Next-generation sequencing technology (NGS) quantitative Real-time polymerase chain reaction (qRT-PCR), Northern Blotting
	cfDNA	N/A	0.5-1.0 µg/mL 52	Diagnose biomarkers, monitor cancer, detect infectious diseases, non-invasive prenatal testing, aging research	Cancer, autoimmune diseases, viral infections	Quantitative polymerase chain reaction (qPCR), next generation sequencing (NGS)
Hormones	Melatonin	0.3-17.6 pg/mL 53	10-80 mg/mL 53	Circadian rhythm regulation, antioxidants, maintain oral health	Sleep disorders, depression, anxiety, seasonal affective disorder, Alzheimer's disease, Parkinson's disease	Enzyme-linked immunosorbent assay (ELISA), radioimmunoassay (RIA), liquid chromatography-mass spectrometry
	Testosterone	Males: 44-148 pg/mL 54 Females: 16-55 pg/mL 55	Males: 3-10 ng/mL 56 Females: 10-80 pg/mL 57	Reflect the endocrine status of the individual, reflects the psychological and behavioral effects	Depression, sexual dysfunction, abnormal growth hormone secretion	Enzyme-linked immunosorbent assay (ELISA), liquid chromatography-tandem mass spectrometry (LC-MS)
	Estradiol	Males: 0.25 to 3.93 pg/mL 58	Males: 10-50 pg/mL	Reflect the endocrine status of the individual, reflects the psychological and behavioral effects	Depression, sexual dysfunction, abnormal growth hormone secretion	Enzyme-linked immunosorbent assay (ELISA), liquid chromatography-tandem mass spectrometry (LC-MS)
		Females: 0.25 to 6.13 pg/mL 58	Females: 15-350 pg/mL			
	Cortisol	0.5-50 µg/mL 59	50-230 ng/mL 60	Evaluate the individual's stress response and circadian rhythm, reflect oral health	Hypoadrenalism, Cushing's syndrome, Addison's disease, polycystic ovary syndrome, depression, anxiety	Enzyme-linked immunosorbent assay (ELISA), liquid chromatography-tandem mass spectrometry (LC-MS)
	Insulin	Fasting state: 57.7-346.2 pg/mL 61	1.93±6.08 mg/ mL 62	Monitor an individual's insulin levels to affect oral health	Diabetes mellitus, obesity, metabolic syndrome, polycystic ovary syndrome	Enzyme-linked immunosorbent assay (ELISA), liquid chromatography-tandem mass spectrometry (LC-MS), radioimmunoassay (RIA)
Metabolites	Uric acid	19 µg/mL 63	Male: 24-60 μg/mL Female: 14-55 μg/mL	Antioxidants, monitor and predict gout and heart disease	Hyperuricemia, gout	Enzyme-linked immunosorbent assay (ELISA), liquid chromatography-mass spectrometry (LC-MS), nuclear magnetic resonance (NMR), biosensors
	Lactic acid	0-280 µg/mL 64	128-968 µg/mL 64	Evaluate the individual's exercise intensity and fatigue levels	Oral cancer, diabetes mellitus, respiratory diseases	Enzyme-linked immunosorbent assay (ELISA), high-performance liquid chromatography (HPLC), mass spectrometry (MS), nuclear magnetic resonance (NMR), biosensors
	Glucose	0.5-1.00 µmol/mL 65	4-6 µmol/mL 65	Monitor the body's metabolism and digestion, and monitor the blood glucose level	Diabetes mellitus, obesity	High-performance liquid chromatography (HPLC), near-infrared spectroscopy (NIRS), Biosensors
Others	Extracellular vesicles	1.11 g/mL 66	1.5 x 10^8^ to 1.5 x 10^9^ particles/mL 67	Transduce signal, early diagnosis, and monitor disease	Oral cancer, diabetes mellitus, HIV, HPV	Flow cytometry (FC), western blotting (WB), mass spectrometry (MS), quantitative Real-time polymerase chain reaction(qRT-PCR)
	Na^+^	11.5-217.3 µmol/mL 68	135-145 µmol/mL 69	Maintain electrolyte balance, promote taste delivery, and have antibacterial effects	Dehydration, xerostomia, renal dysfunction, endocrine disorders	Ion-selective electrode (ISE), inductively coupled plasma mass spectrometry (ICP-MS), ion chromatography (IC)
	K^+^	2.6-18.3 µmol/mL 68	3.5-5.0 µmol/mL 70	Maintain ion balance inside and outside cells, protect teeth and oral tissues, participate in nerve signaling	Dehydration, sialadenitis, renal dysfunction, endocrine disorders	Ion-selective electrodes (ISE), atomic absorption spectrometry (AAS), atomic emission spectrometry (AES), near-infrared spectroscopy (NIRS), biosensors
	Cl^-^	0.5-3.5 µmol/mL 71	97-107 µmol/mL 72	Maintain acid-base balance, combine with peroxidase to act as an antimicrobial	Dehydration, cystic fibrosis, Sjogren syndrome	Ion selective electrode (ISE), high-performance liquid chromatography (HPLC), atomic emission spectrometry (AES)
	Ca^2+^	1-4 µmol/mL 73	2.2-2.65 µmol/mL 74	Enamel mineralization, anti-caries effect, maintain electrolyte balance, influence saliva viscosity	Periodontal disease, xerostomia	Atomic absorption spectrometry (AAS), ion selective electrode (ISE), inductively coupled plasma mass spectrometry (ICP-MS)
	PO_4_^3-^	5-35 μg/mL 75	25-45 μg/mL 76	Tooth mineralization, anti-caries effect, maintain oral pH	Parathyroid dysfunction, osteoporosis, diabetes mellitus	Spectroscopic, and chromatographic methods
	HCO_3_^-^	1-60 µmol/mL 71	22-26 µmol/mL 77	Prevents acid damage, maintains mouth pH, and facilitates digestion	Dental caries, periodontal disease, reflux esophagitis, sjogren syndrome, metabolic acidosis	Ion-selective electrode (ISE), pH meter
	Mg^2+^	2.81-3.61 mg/mL 78	0.7-1 μmol/mL 79	Enamel heavy mineralization, stabilize activating enzymes, stabilize nucleic acids	Diabetes mellitus, renal dysfunction	Atomic absorption spectroscopy (AAS), Ion-selective electrodes (ISE)
	NH_3_	4.4 µmol/mL 80	21-57 µmol/mL 80	Maintain oral pH, bacterial metabolites	Kidney dysfunction, digestive system diseases	Gas chromatography (GC), ion chromatography (IC)

**Table 2:** Comparison of stimulated saliva collection and unstimulated saliva collection.

Collection Type	Methodology	Collection Volume	Collection Time	Impact on Detection	Post-Collection Processing Difficulty	References
Unstimulated Saliva Collection	Parotid gland using cotton swab.	Low	Long	Minimizes analyte dilution, better basal state representation.	Potentially high due to filtration	132,133
	Submandibular/sublingual glands using syringe every second minute.	Moderate	Moderate	Better basal state representation.	Moderate	134
Stimulated Saliva Collection	Parotid gland using polyethylene tubes or cone-shaped sialographic cannulas.	High	Long	Suitable for clinical diagnosis, less contamination.	High due to invasive methods	135,136
	Parotid gland using Carlsson-Critten collector or Lashley cup with citric acid stimulation.	High	Moderate	Suitable for clinical diagnosis, less contamination.	Moderate	135,137
	Whole saliva via chewing gum or tasting.	High	Short to moderate	Increased salivary flow and pH; variable composition based on stimulation method.	Low	138,139 140,133

**Table 3:** Comparison of different saliva processing methods.

Methods		Advantages	Disadvantages	Biomarkers	Detection techniques
Physical treatment	Centrifugation	Remove large particles of impurities and cells.	Lose some small molecules and a high demand for saliva.	Total protein concentration, enzymes (e.g., amylase)	Enzyme-linked immunosorbent assay (ELISA), spectrophotometry
	Filtration	Remove large particles of impurities and reduces the number of cells and impurities in the sample.	Lose of some target analytes.	Cytokines (e.g., IL-6, IL-8), microRNA.	ELISA, PCR, RT-PCR.
	Cryopreservation	Preserve samples for long periods without loss of quality.	Introduce damage to the sample from ice crystals, affecting subsequent analysis.	Long-term storage of DNA, RNA, and proteins.	Freezing, lyophilization, cryopreservation.
	Centrifugal microfluidics	Low consumption of samples and reagents, rapid mixing, separation, and reaction; high level of system integration, easy reading and analysis of test results.	Factors affecting sample pre-treatment, chip clogging, contamination, design restrictions, initial chip series costs, and performance sensitivity to flow conditions include complexity and potential issues.	Multiplex assays, point-of-care testing, proteomics.	Microfluidic chips, lab-on-a-chip, biosensors.
**Methods**		**Advantages**	**Disadvantages**	**Biomarkers**	**Detection techniques**
Chemical treatment	Addition of SDS	Release tiny molecules; interact with positively charged biomolecules and aid in their separation; dissolve and destroy big molecules (such as proteins and lipids).	Introduction of impurities.	Protein profiling, enzyme activities.	SDS-PAGE, Western blotting, mass spectrometry.
	Adjustment of PH	Quickly reduce protein interference by altering biomolecule solubility and charge;dissolve calcium phosphate crystals to prevent interference;increase the relative abundance of target analytes to increase detection sensitivity.	Dissolve or destroy some small molecules; cause side reactions and unreliable analysis results.	Salivary pH measurement, ionized biomolecules, enzyme activities.	pH meter, ion-selective electrodes, spectrophotometry.
	Preservative method	Inhibits microorganism growth; prevents enzymatic reactions; maintains sample integrity.	Preservatives vary in requirements for different samples; choice is limited; some preservatives can corrode equipment and containers.	Microbial DNA/RNA, proteins, enzymes.	PCR, ELISA, next-generation sequencing (NGS).

## Abbreviations

Evs: extracellular vesicles
HTS: high-throughput sequencing
EFIRM: electric field-induced release and measurement
ctDNA: tumor DNA
EGFR: epidermal growth factor receptor
NSCLC: non-small cell lung cancer
ELISA: enzyme-linked immunosorbent assay
MS: mass spectrometry
WB: western blotting
LC-MS: liquid chromatography-tandem mass spectrometry
qRT-PCR: quantitative real-time polymerase chain reaction
IF: immunofluorescence
HPLC: high-performance liquid chromatography
NMR: nuclear magnetic resonance spectroscopy
2-DE: 2D gel electrophoresis
RNA-Seq: RNA sequencing
NGS: next-generation sequencing technology
RIA: radioimmunoassay
NIRS: near-infrared spectroscopy
FC: flow cytometry
ISE: ion-selective electrode
ICP-MS: inductively coupled plasma mass spectrometry
IC: ion chromatography
AAS: atomic absorption spectrometry
AES: atomic emission spectrometry
GC: gas chromatography
MMPs: matrix metalloproteinases
GC-MS: gas chromatography-mass spectrometry
OSCC: oral squamous cell carcinoma
OPMD: oral potentially malignant disorders
PSA: prostate-specific antigen
cfRNA: cell-free RNA
BRCA: breast cancer gene
hCG: human chorionic gonadotropin
UA: uric acid
HNSCC: head and neck squamous cell carcinoma
CTCs: circulating tumor cells
NDDs: neurodegenerative disorders
HO-1: heme oxygenase-1
SDS: sodium dodecyl sulfate
WTD: wooden tongue depressor
SPCE: screen-printed carbon electrode
μPAD: microfluidic paper-based analytical device
LOD: limit of detection
LOQ: limit of quantification
DSS: dried saliva spot
